# *Nocardia cyriacigeorgica* Identified by Bronchial Washing During Evaluation of Radiologic Progression in a Patient With Prior Nontuberculous Mycobacterial Diseases

**DOI:** 10.1002/rcr2.70654

**Published:** 2026-07-19

**Authors:** Hidenori Takahashi, Kanako Furukawa, Yugo Satake, Natsuko Nakano, Shinichiro Ota, Masaharu Shinkai

**Affiliations:** ^1^ Department of Respiratory Medicine Tokyo Shinagawa Hospital Tokyo Japan; ^2^ Department of Pathology Tokyo Shinagawa Hospital Tokyo Japan

**Keywords:** bronchial washing, bronchiectasis, *Nocardia cyriacigeorgica*, nontuberculous mycobacteria, pulmonary nocardiosis

## Abstract

Pulmonary nocardiosis may occur in immunocompetent patients with structural lung disease and may resemble the progression of nontuberculous mycobacterial pulmonary disease. We report an 81‐year‐old immunocompetent woman with chronic bronchiectasis and prior *Mycobacterium kansasii* , *M. intracellulare* , and *M. abscessus* infections. Despite persistently negative sputum mycobacterial cultures, chest computed tomography (CT) revealed progressive nodular bronchiectatic changes. Bronchial washing from the right B5 bronchus yielded *Nocardia cyriacigeorgica* ; mycobacterial studies were negative. Trimethoprim–sulfamethoxazole was stopped shortly after initiation because of drug eruption, and minocycline monotherapy led to transient symptomatic and radiologic improvement. However, follow‐up CT at 6 months revealed radiologic progression. Repeat bronchoscopy was culture‐negative, but bronchial washing cytology revealed filamentous organisms suggestive of persistent nocardial infection. Minocycline monotherapy was continued for 12 months. Chest CT at 12 months revealed new contralateral left upper lobe bronchocentric abnormalities. This case highlights the importance of bronchial washing and longitudinal reassessment when radiologic progression remains unexplained by sputum evaluation.

## Graphical abstract

In this case of pulmonary nocardiosis complicating prior nontuberculous mycobacterial pulmonary disease, repeat bronchial washing cytology revealed filamentous organisms suggestive of ongoing nocardial infection despite negative cultures. Bronchoscopic sampling may therefore aid in not only initial diagnosis but also reassessment when sputum studies are negative or unobtainable.

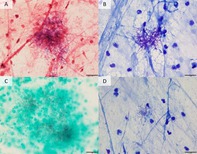

## 1 Introduction

Pulmonary nocardiosis is classically regarded as an opportunistic infection in immunocompromised individuals; however, it can also occur in immunocompetent patients with chronic structural lung diseases such as bronchiectasis [1, 2]. In patients with a history of nontuberculous mycobacterial pulmonary disease (NTM‐PD), nocardial infections may coexist with NTM or emerge during the clinical course [3, 4]. Consequently, radiologic progression may closely resemble NTM relapse or worsening, and attributing imaging progression solely to NTM can lead to delayed diagnosis and inappropriate therapy [1, 2, 3, 4]. Herein, we report the case of an immunocompetent older woman with a long history of NTM disease and treatment, in whom bronchoscopy with bronchial washing enabled the microbiological identification of *Nocardia cyriacigeorgica* and informed subsequent clinical decision‐making.

## 2 Case Report

An 81‐year‐old never‐smoking woman with chronic bronchiectasis predominantly involving the right middle lobe and lingula presented with fever, chills, and blood‐tinged sputum. She regularly engaged in gardening and had no history of immunodeficiency or immunosuppressive therapy. The patient had a 10‐year history of serial NTM isolation and management. *Mycobacterium kansasii* infection had previously been treated with isoniazid, rifampin, and ethambutol for 14 months. *Mycobacterium intracellulare* was subsequently isolated and managed with close clinical follow‐up without antimicrobial therapy. She later developed *Mycobacterium abscessus* infection, for which she received imipenem/cilastatin, amikacin, and clarithromycin, followed by maintenance therapy with clarithromycin plus faropenem.

Despite persistently negative sputum cultures for mycobacteria over the previous year, chest computed tomography (CT) revealed radiologic progression (Figure 1A). Diagnostic bronchoscopy was performed to investigate the cause of this progression, and bronchial washing was obtained from the right B5 bronchus using 20 mL of sterile saline.

**FIGURE 1:**
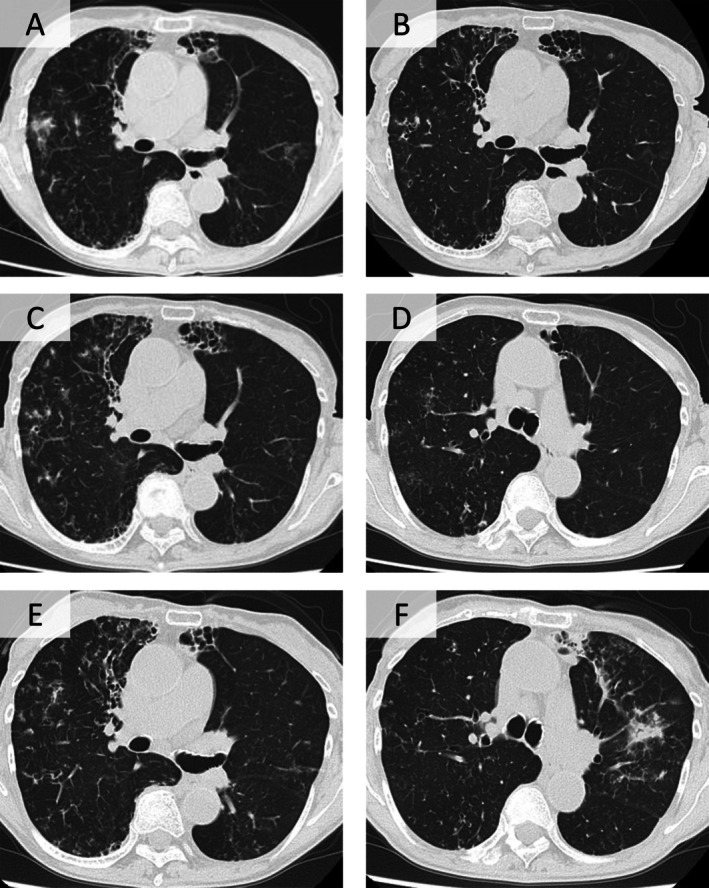
Serial chest CT findings. (A) CT at presentation showing chronic right middle lobe/lingular bronchiectasis with bronchial wall thickening, accompanied by increased centrilobular nodules and branching tree‐in‐bud opacities (right‐predominant), consistent with active endobronchial spread. (B) Chest CT at 2 months after initiating anti‐nocardial antimicrobial therapy showing reduced centrilobular nodules/tree‐in‐bud opacities and peribronchial inflammatory changes. (C) Chest CT at 6 months showing recurrent and increased tree‐in‐bud opacities and centrilobular nodules in the right lung, consistent with radiologic re‐progression. (D) Chest CT at 6 months showing no definite bronchocentric abnormality in the left upper lobe at this level. (E) Chest CT at 12 months showing mixed interval changes in the right lung lesions, with partial improvement in some areas and worsening in others. (F) Chest CT at 12 months showing new left upper lobe bronchocentric abnormalities at a level comparable to that in panel D, including bronchiectatic/bronchiolectatic changes, bronchial wall thickening, peribronchial patchy consolidation, and surrounding centrilobular nodules/tree‐in‐bud opacities.

Mycobacterial smears, cultures, and polymerase chain reactions of the bronchial washing fluid were negative. *N. cyriacigeorgica* was isolated from the bronchial washing specimens; no other significant bacterial or fungal pathogens were identified.

Antimicrobial susceptibility testing revealed susceptibility to trimethoprim–sulfamethoxazole (TMP–SMX); intermediate susceptibility to minocycline; and resistance to clarithromycin, faropenem, and fluoroquinolones (Table 1). Head CT revealed no evidence of intracranial involvement. Given the negative mycobacterial results, pulmonary nocardiosis was considered the most likely explanation for the radiologic progression, and treatment was modified accordingly. Clarithromycin and faropenem were discontinued, and TMP–SMX was initiated.

**TABLE 1:** Antimicrobial susceptibility profile of *Nocardia cyriacigeorgica* isolated from bronchial washing fluid.

Antimicrobial agent	MIC (μg/mL)	Interpretation
Faropenem	—	R
Doxycycline	—	I
Penicillin G	> 2	R
Cefotaxime	32	I
Ceftriaxone	32	I
Cefepime	> 32	R
Imipenem	2	S
Amikacin	≦ 4	S
Erythromycin	> 4	R
Clarithromycin	—	R
Clindamycin	> 2	R
Minocycline	2	I
Linezolid	≦ 1	S
Levofloxacin	—	R
Ciprofloxacin	> 2	R
Moxifloxacin	—	R
Sulfamethoxazole/trimethoprim	20	S

*Note:* MICs and interpretive categories are shown for the isolate recovered at the initial bronchoscopy.

Abbreviations: I, intermediate; MIC, minimum inhibitory concentration; R, resistant; S, susceptible.

After 10 days of TMP–SMX therapy, the patient developed an erythematous eruption. TMP–SMX was suspected to be the causative drug and therefore discontinued. Topical corticosteroid therapy led to resolution of the rash. TMP–SMX desensitization was discussed; however, the patient declined this approach. Therefore, minocycline (200 mg/day) was initiated as an alternative therapy. Her presenting symptoms subsequently improved, and sputum production was reduced.

Follow‐up CT at 2 months showed improvement (Figure 1B), but CT at 6 months demonstrated interval radiologic progression of the pulmonary lesions (Figure 1C,D). As the sputum was scant and could not be obtained for microbiological evaluation, repeat bronchoscopy was performed to obtain bronchial washing from the right B5 bronchus in order to clarify the discrepancy between imaging and microbiological assessment. Cultures of the bronchial washing fluid were negative for routine bacterial pathogens, mycobacteria, fungi, and *Nocardia*; however, cytologic examination revealed aggregates of filamentous bacteria that were positive on Gram staining, Kinyoun staining, and Grocott staining, and weakly positive on Ziehl–Neelsen staining, findings suggestive of persistent nocardial infection (Figure 2A–D).

**FIGURE 2:**
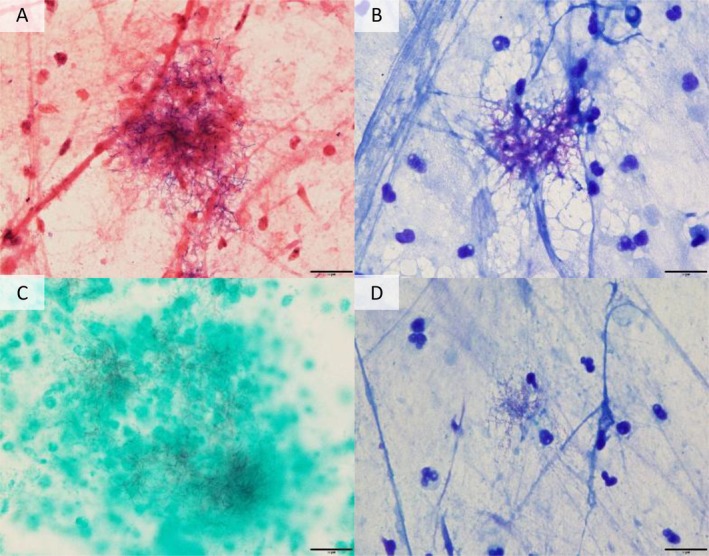
Cytology findings of bronchial washing obtained at the time of radiologic re‐progression. (A) Gram stain shows tangled aggregates of slender, branching, beaded Gram‐positive filaments in an inflammatory background. (B) Modified acid‐fast (Kinyoun) demonstrates partial/weak acid‐fastness. (C) Grocott methenamine silver (GMS) highlights the filamentous organisms. (D) Ziehl–Neelsen shows faint acid‐fast staining, supporting possible persistent nocardial infection. (Scale bars as indicated).

Because no culture growth of *Nocardia* was obtained despite cytologic findings suggestive of possible persistence, immediate escalation of anti‐nocardial therapy was not pursued at that time. More intensive anti‐nocardial therapy, including intravenous imipenem or amikacin, linezolid, TMP–SMX desensitization, or combination therapy, was considered; however, minocycline monotherapy was continued during outpatient follow‐up until 12 months after initiating anti‐nocardial treatment.

During follow‐up, the patient exhibited intermittent fever, and serum C‐reactive protein levels fluctuated between 0.02 and 1.32 mg/dL. Sputum was not expectorated during this period; therefore, further sputum cultures could not be performed. Twelve months after initiating anti‐nocardial therapy, CT revealed partial improvement in the right lung lesions along with new left upper lobe bronchocentric abnormalities, including bronchiectatic/bronchiolectatic changes, bronchial wall thickening, peribronchial patchy consolidation, and surrounding centrilobular nodules/tree‐in‐bud opacities (Figure 1E,F). Overall, the 12‐month CT findings did not provide sufficient evidence for sustained disease control with minocycline monotherapy. The patient remains under outpatient follow‐up, with continued clinical and radiologic monitoring.

## 3 Discussion

This case highlights the diagnostic challenges in patients with chronic NTM‐related bronchiectatic disease. A nodular bronchiectatic pattern is well recognized in NTM‐PD; however, pulmonary nocardiosis can present with symptoms similar to those of NTM‐PD, particularly in immunocompetent patients [2, 5]. Pulmonary nocardiosis should be considered in patients with a history of NTM‐PD and worsening CT abnormalities, particularly when the results of repeat mycobacterial studies are negative or nondiagnostic [2, 3, 4, 5]. In some cases, *Nocardia* is detected only via bronchoscopic sampling when sputum‐based evaluation is nondiagnostic [3, 4]. In this context, bronchial washing in the present case was informative as it enabled species‐level identification during the initial bronchoscopy, and cytology provided supportive evidence for re‐evaluation despite negative repeat cultures [6]. This is clinically relevant because culture negativity may occur after antimicrobial exposure or when the microorganism burden is low.

*N. cyriacigeorgica* is a common clinical *Nocardia* species that remains susceptible to TMP–SMX, although its minocycline susceptibility is less reliable [7]. In the present case, TMP–SMX was discontinued because of drug eruption, and minocycline was used as an alternative despite its intermediate susceptibility. The initial symptomatic improvement was followed by intermittent fever along with elevated serum C‐reactive protein levels, radiologic progression during follow‐up, and subsequent development of new contralateral bronchocentric abnormalities. Collectively, these findings suggest that minocycline monotherapy was insufficient to achieve sustained disease control. The effects of alternative anti‐nocardial regimens could not be assessed because no intensified regimen was introduced. The present case underscores the importance of assessing treatment response in such complex airway infections by integrating symptoms, inflammatory markers, imaging findings, and microbiologic data [8].

These new abnormalities highlighted the site‐specific nature of bronchoscopic sampling. As the first bronchial washing had been obtained only from the right B5 lesion, the microbiologic basis of the new left‐sided lesions could not be determined from the earlier bronchoscopic results. This distinction is important because bilateral or multifocal bronchocentric abnormalities may reflect NTM‐PD, pulmonary nocardiosis, or other airway infections [1, 2, 8]. In conclusion, this case illustrates that pulmonary nocardiosis can mimic progression of NTM‐PD, while recurrent or concomitant NTM disease may remain difficult to exclude, underscoring the importance of longitudinal reassessment in such complex cases.

## Author Contributions

Conceptualization, methodology, investigation, writing – original draft preparation, and project administration: H.T. Resources and visualization: H.T. and N.N. Writing – review and editing: K.F., Y.S., N.N., S.O., and M.S. All authors have read and agreed to the final version of the manuscript.

## Funding

The authors have nothing to report.

## Ethics Statement

All procedures were performed in compliance with relevant laws and institutional guidelines.

## Consent

The authors declare that written informed consent was obtained for the publication of this manuscript and accompanying images using the consent form provided by the Journal.

## Conflicts of Interest

The authors declare no conflicts of interest.

## Data Availability

The data that support the findings of this study are available on request from the corresponding author. The data are not publicly available due to privacy or ethical restrictions.
